# Raltegravir-Induced Adaptations of the HIV-1 Integrase: Analysis of Structure, Variability, and Mutation Co-occurrence

**DOI:** 10.3389/fmicb.2019.01981

**Published:** 2019-09-03

**Authors:** Lucas de Almeida Machado, Marcelo Ferreira da Costa Gomes, Ana Carolina Ramos Guimarães

**Affiliations:** ^1^Laboratory for Functional Genomics and Bioinformatics, Instituto Oswaldo Cruz, Oswaldo Cruz Foundation (Fiocruz), Rio de Janeiro, Brazil; ^2^Scientific Computing Program, Oswaldo Cruz Foundation (Fiocruz), Rio de Janeiro, Brazil

**Keywords:** HIV-1, integrase, raltegravir, resistance, entropy, co-occurrence, mutation

## Abstract

The human immunodeficiency virus type 1 (HIV-1) has several proteins of therapeutic importance, many of which are currently used as drug targets in antiretroviral therapy. Among these proteins is the integrase, which is responsible for the integration of the viral DNA into the host genome – a crucial step for HIV-1 replication. Given the importance of this protein in the replication process, three integrase inhibitors are currently used as an option for antiretroviral therapy: Raltegravir, Elvitegravir, and Dolutegravir. However, the crescent emergence of mutations that cause resistance to these drugs has become a worldwide health problem. In this study, we compared the variability of each position of the HIV-1 integrase sequence in clinical isolates of Raltegravir-treated and drug-naïve patients by calculating their Shannon entropies. A co-occurrence network was created to explore how mutations co-occur in patients treated with Raltegravir. Then, by building tridimensional models of the HIV-1 integrase intasomes, we investigated the relationship between variability, architecture, and co-occurrence. We observed that positions bearing some of the major resistance pathways are highly conserved among non-treated patients and variable among the treated ones. The residues involved in the three main resistance-related mutations could be identified in the same group when the positions were clustered according to their entropies. Analysis of the integrase architecture showed that the high-entropy residues S119, T124, and T125, are in contact with the host DNA, and their variations may have impacts in the protein-DNA recognition. The co-occurrence network revealed that the major resistance pathways N155H and Q148HR share more mutations with each other than with the Y143R pathway, this observation corroborates the fact that the N155H pathway is most commonly converted into Q148HRK than into Y143RCH pathway in patients’ isolates. The network and the structure analysis also support the hypothesis that the resistance-related E138K mutation may be a mechanism to compensate for mutations in neighbor lysine residues to maintain DNA binding. The present study reveals patterns by which the HIV-1 integrase adapts during Raltegravir therapy. This information can be useful to comprehend the impacts of the drug in the enzyme, as well as help planning new therapeutic approaches.

## Introduction

The human immunodeficiency virus type 1 (HIV-1) is a Lentivirus from the *Retroviridae* family and one of the causative agents of Acquired Immunodeficiency Syndrome (AIDS) along with HIV-2 (Marlink et al., 1994; Freed, 2001). HIV-1 has many proteins responsible for important replication steps, many of which are currently used as targets for the antiretroviral therapy (Yarchoan et al., 1991; Markowitz et al., 2007; Hare et al., 2010; World Health Organization, 2010), and the integrase (IN) is among the main targets (Fesen et al., 1993). The IN is a 288 residue protein responsible for the integration of the viral DNA (vDNA) into the host DNA (tDNA) strand, a crucial step for HIV-1 replication (Blanco et al., 2011). This protein can be divided into three functional domains: the N-terminal domain (NTD), the catalytic core domain (CCD) and the C-terminal domain (CTD) connected by short linker regions (Passos et al., 2017). To integrate the vDNA into the host genome, IN catalyzes two reactions: the 3′-processing, in which two or three nucleotides are removed from 3′ ends of the vDNA; and the strand transfer reaction, in which the processed 3′ ends of the vDNA are inserted into the tDNA (Blanco et al., 2011).

The only class of IN inhibitors clinically available for therapy are the Integrase strand transfer inhibitors (INSTI) that impair the strand transfer reaction. Raltegravir (RAL) was the first INSTI widely used, followed by Elvitegravir (EVG), constituting the first generation INSTIs, recently the only second generation INSTI, Dolutegravir (DTG), was approved (Fesen et al., 1993; Evering and Markowitz, 2007; Shimura and Kodama, 2009; Blanco et al., 2011; Pendri et al., 2011; Akil et al., 2015).

After the first years of clinical use of RAL, many resistance-related mutations emerged (Charpentier et al., 2008; Cooper et al., 2008; Miller et al., 2008; Malet et al., 2009). Among the ten major resistance-related positions listed in the HIVdb – positions 66, 92, 118, 138, 140, 143, 147, 148, 155, 263 (Shafer, 2006), the residues Y143, Q148, and N155 are the hotspots with the highest number of resistance-related mutations documented according to the HIVdb (Shafer, 2006), being the three main resistance-related positions. In the early stages of therapy, the mutation N155H tends to appear and can be later substituted by either the Y143R or Q148HKR pathway after prolonged treatment (Johnson et al., 2007; Charpentier et al., 2008; Fransen et al., 2008; Miller et al., 2008; Malet et al., 2009; Quercia et al., 2009). The mutations in the three main resistance-related positions are mutually exclusive; however, it was also shown that many different isolates bearing different resistance pathways could coexist in the same patient (Charpentier et al., 2008). Some of the major resistance polymorphisms can be associated with accessory mutations, which compensate for the loss of function caused by the primary mutations (Quercia et al., 2009).

Structural information about the interaction between the IN, vDNA, and tDNA is crucial to comprehend the features that determine the selection patterns for resistance-related mutations. Recently, the structure of the tetrameric complex of the IN bound to vDNA and tDNA after the strand transfer reaction (the so-called strand transfer complex – STC), was determined by cryogenic electron microscopy (cryo-EM) (Passos et al., 2017). The STC is comprised of two inner chains (A and C), which are in contact with the vDNA molecule, and two outer chains (B and D), contacting the tDNA. A and C form dimers, respectively with B and D. A previous study (Ceccherini-Silberstein et al., 2009) showed the relationship between sequence variation and the IN structure; however, by the time this study was published, there was no IN structure available showing interaction between the tetramer and the DNA. Additionally, another recent study analyzed the IN variations, focusing on the structural implications of some of the HIV-1 integrase mutations (Rogers et al., 2018). Here we evaluated the IN variability caused by RAL treatment by comparing the Shannon entropy of each position of the IN sequence in drug-naïve and treated patients. Using a co-occurrence network, we also analyzed the patterns by which mutation pairs occur in isolates from RAL-treated patients. Finally, we constructed tridimensional models of the STC, as well as the complex before the strand transfer reaction (the cleaved stable synaptic complex – cSSC), to study the structural patterns that guide variability. Data on entropy, co-occurrence, and structure were compared, to shed light into the patterns by which the IN evolves during RAL treatment.

## Materials and Methods

### Dataset

The first step to build the co-occurrence network and calculate the Shannon entropies was building a dataset of sequences of the HIV-1 IN from isolates of drug-naïve patients and RAL-treated patients deposited on the HIVdb (Shafer, 2006). Only full-length sequences of the subtype B with no nucleotide ambiguities and deposited since 2007 were included in the dataset. The sequences of the isolates were translated to amino acid sequences and aligned to the IN reference sequence (UniProt (Chen et al., 2011) entry Q76353) using the MUSCLE algorithm (Edgar, 2004) to characterize the polymorphisms.

### Shannon Entropy

To assess the variability in each position of the IN sequence, we used the Shannon entropy (Shannon, 1948), which measures the amount of information in a set of data. In this specific case, the amount of information in a multiple-sequence alignment column. Columns with low Shannon entropy indicate low variability in that given position, whereas higher entropy values point otherwise. Given that the main resistance-related mutations are shown to impact the strand transfer function (Marinello et al., 2008), these positions are expected to be conserved in drug-naïve patients – therefore showing low entropies within the naïve patients’ dataset – and more diverse in RAL-treated patients – thus, expected to show higher entropies in the RAL-treated patients’ dataset.

The Shannon entropy was calculated according to Eq. 1. Where *i* represents the index of the columns in a multiple sequence alignment, and *P*(*r*_*j*_) represents the probability of finding an amino acid *j* in column *i*, given that *j* iterates from 1 to 20, where each index of *j* represents one of the 20 common amino acids.

(1) $$H_{i}=-\sum_{j=1}^{20}P\,\left(r_{j}\right)\,{\text{log}}_{10}\,P\,\left(r_{j}\right)$$

To calculate the Shannon entropy of the RAL-treated patients (H_RAL_) and the naïve patients (H_na__ï__ve_) for each dataset, we used a bootstrap method. To do so, 1000 random samples of 50 sequences were taken from each dataset. Multiple sequence alignments were performed in each sample using the MUSCLE algorithm, and the Shannon entropy was calculated for each position in the alignments. Following this, the average entropies of all the multiple-sequence alignments of the drug-naïve and RAL-treated patients were calculated, as well as the standard deviations. To understand the variability patterns, the positions were clustered by their H_na__ï__ve_ and H_RAL_ using a hierarchical clustering algorithm (McQuitty, 1966) and divided into four clusters.

### Co-occurrence Network

From the list of mutations found in the isolates of RAL-treated patients, a square matrix was built, where the rows and columns represent the mutations observed in the dataset. In this analysis, we only considered polymorphisms that had frequencies within the 0.75 quantile, to exclude underrepresented mutations. Each element of the matrix contained the co-occurrence index (ξ) of its pair of mutations *ij*. The ξ value is given by the Jaccard index of the correspondent mutation pair (Eq. 2). γ_AB_ is the number of sequences that have mutation A and mutation B. This value is divided by the number of all sequences that have mutation A or mutation B. The resulting ξ index ranges from 0 to 0.5, where 0.5 means that every sequence that has mutation A also has mutation B and vice-versa; whereas a ξ index equals to zero means that the pair of mutations never appear together in any sequence.

(2) $$\xi =\frac{{\text{γ}}_{A\,B}}{A+B}$$

The co-occurrence matrix was then used as an adjacency matrix to build a non-directed weighted network. The nodes of the network represent mutations connected by their ξ values. To exclude noise from pairs with low co-occurrence indexes and remove noise, only mutation pairs with ξ indexes greater than 0.1 were considered when the network was built.

To analyze the clustering structure of the network, the mutations were clustered using the Markov Clustering Algorithm (MCL), which applies a random walk simulation, clustering the nodes in communities were the information tends to be contained (Dongen, 2000).

### Comparative Modeling

To understand the structural features of each position of the HIV-1 integrase, we constructed tridimensional models for two states of the protein complex: the cSSC – which is the IN tetramer bound to the vDNA – and the STC – which is the tetramer of IN bound to vDNA and tDNA after the strand transfer reaction. To construct the structures, we used the consensus sequence of the HIV-1 integrase of subtype B (UniProt accession B9VIC1), and the models were generated with the software MODELLER 9.18. One of the templates used in comparative modeling was the HIV-1 STC structure obtained by cryo-EM (Passos et al., 2017). This cryo-EM structure is a large tetrameric complex that surrounds the vDNA already bound to tDNA. Since the structure lacks coordinates for residues 205–222 of the inner chains and residues 187–217 from the outer chains, we also used a crystal structure of the HIV-1 integrase available on the PDB (1ex4) (Chen et al., 2000) as a template. The cryo-EM STC structure also lacks the coordinates of one of the two Mg^2+^ ions on the active site. Therefore, we aligned the structure with the prototype foamy virus (PFV) intasome structure (3OYA) (Hare et al., 2010) and used the coordinates of its Mg^2+^ ions. The two inner chains (close to the vDNA) were modeled from residue 1 to residue 269, and the outer chains were modeled from residue 58 to 269 – since the cryo-EM structure lacks the C-terminal tail of all chains and the N-terminal of the outer chains. For modeling the cSSC structure, the region corresponding to the tDNA was not considered. Twenty models were generated for each system. To build each model, we used an optimization protocol of 300 iterations of energy minimization by conjugate gradient with modeller’s variable target function method, followed by modeller’s molecular dynamics routine. For each model, the optimization was repeated at least two times or until the molpdf (modeller probability density function) returned values greater than 1 × 10^6^. The model with the lowest DOPE score of each system was validated by inspection of its stereochemical properties, and used for the analysis.

## Results

### Dataset

The final dataset contained 158 sequences from RAL-treated patients and 1166 sequences from drug-naïve patients (available in the Supplementary Material).

Curiously, four isolates showed mutations related to more than one of the three main resistance pathways: two from Italy (Canducci et al., 2010), one with both N155H and Y143R and another with Q148H and Y143H; one from Canada (Brenner et al., 2011) with N155H and Y143C; and one from France with N155H and Q148R. The last combination, however, was previously described in *in vitro* experiments as having resistance levels so high that could not be measured within the range of RAL used on the assays and was never found *in vivo* (Fransen et al., 2008). Nevertheless, Malet et al. (2009), isolated this clone bearing both polymorphisms. The frequency of the mutations present in the RAL-treated patients can be seen in the Supplementary Table S1. Interestingly, none of the mutations in the three main resistance-related positions are present in more than 30% of the isolates. It may reflect the fact that the dataset contains patients in different treatment stages. It is important to note that in our dataset of RAL-treated patients we did not find any mutation in positions 51, 114, 121 and 149, which are known for bearing resistance-related mutations. Also, in position 66, we only found the T66K variant.

### Shannon Entropy

The Shannon entropies calculated from the bootstrap for each sequence position showed values between zero and 1.7 bits. The maximum standard deviation found among the entropies was of 0.15 bits, which shows consistency within the datasets.

**FIGURE 1:**
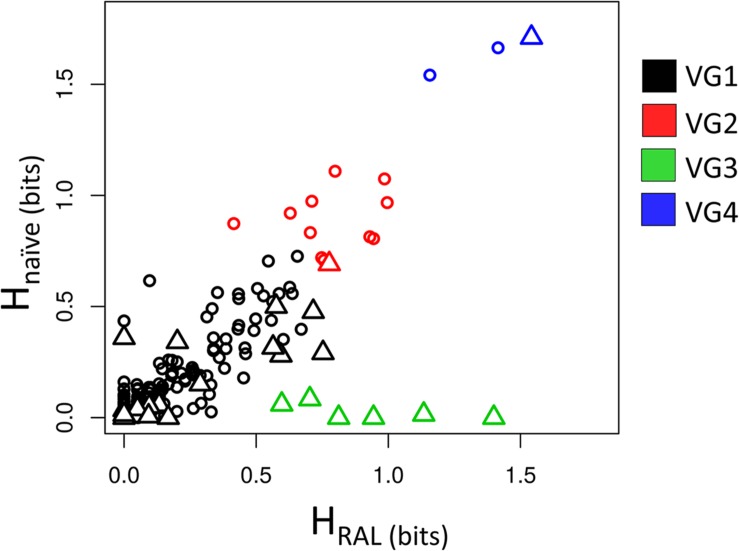
Shannon entropy of RAL-treated patients vs. drug-naïve patients. The scatter plot shows the correspondence between H_RAL_ and H_na__ï__ve_. Positions are represented as triangles if there is any empirical evidence of its role in resistance to INSTIs; and circles, if there is no evidence for implications in resistance. Each color of the triangles or circles indicates its variability group (VG).

Figure 1 shows the values of H_RAL_ and H_na__ï__ve_. The positions were divided into four variability groups (VG1, VG2, VG3, and VG4). H_RAL_ and H_na__ï__ve_ have a strong correlation (*R* = 0.93). However, there are some outlier positions below the tendency line, as seen in VG3 (represented in Figure 1 in green).

These positions show high entropy in treated patients and low entropy in drug-naïve ones, which may indicate selective pressure in RAL-treated patients, and, as expected, these positions are confirmed as having implications in resistance. Positions 140, 143, 148 and 155 (involved in major resistance pathways (Cooper et al., 2008; Fransen et al., 2008; Blanco et al., 2011), are in VG3, as well as position 138, which may display the resistance mutations E138KAT (Da Silva et al., 2010; Blanco et al., 2011; Rhee et al., 2016). Position 97, which is known for bearing the T97A accessory mutation (Fransen et al., 2008; Eron et al., 2012), is also in this variability group.

Residues 119, 124, and 125 (present in VG4 and represented in blue in Figure 1) are highly variable in treated and naïve patients. In 119, the natural polymorphism S119R is described as weakly selected in RAL-treated patients (Hachiya et al., 2015). It is also important to note that its neighbor G118 may show the mutation G118R (Malet et al., 2011). This polymorphism is selected in DTG-treated patients (Kobayashi et al., 2011) [and in one patient in RAL regime (Malet et al., 2011)]. G118R is considered an accessory mutation and shows a significant reduction in RAL susceptibility (Kobayashi et al., 2008). However, it is not clear how the presence of G118R affects the variability in the neighbor S119.

VG2 cluster (represented in red in Figure 1) has residues that are moderately variable in both datasets. Position 50 is the only resistance-related position in this group. This residue is involved in resistance to DTG when bearing the M50I mutation in combination with R263K and is selected *in vitro* by DTG treatment (Quashie et al., 2012; Wares et al., 2014; Tsiang et al., 2016).

The last cluster, VG1 (represented in black in Figure 1), has positions that have low entropies in both datasets, i.e., regions whose variability is supposedly mildly affected by treatment. However, some resistance-related positions appear in this group. Among them, positions 51 and 163 are known for having accessory mutations (Gatell et al., 2010; Hatano et al., 2010; Blanco et al., 2011; Margot et al., 2012). The residue 92, may show the mutation E92Q that was shown to reduce RAL susceptibility (Jones et al., 2009; Kobayashi et al., 2011). While mutation F121Y, in spite of being rarely selected *in vivo* (Cavalcanti et al., 2012), is capable of lowering RAL efficacy (Kobayashi et al., 2008; Shimura et al., 2008). Both positions 92 and 121 are found in VG1. Position 66 – which is one of the major resistance-related positions – and positions 142 – also associated with resistance-, are also in VG1. T66I reduces EVG susceptibility and has minimal effects over RAL, while T66K reduces both RAL and EVG susceptibility (Charpentier et al., 2008; Shimura et al., 2008; Gatell et al., 2010; McColl and Chen, 2010; Hurt et al., 2013). The rare P142T mutation was reported as selected *in vitro* by DTG (Oliveira et al., 2015) and *in vivo* by RAL (Naeger et al., 2016). L74, V151, and G163 (Cooper et al., 2008; Kobayashi et al., 2008, 2011; Jones et al., 2009; Blanco et al., 2011), which are known for displaying accessory mutations, also appear in VG1. Furthermore, position 230, which may bear the mutation S230R in patients treated with RAL, EVG, or DTG also displays low entropies in both treated and non-treated patients. However, this mutation does not seem to reduce RAL susceptibility (Goethals et al., 2008; Underwood et al., 2015; Pham et al., 2018). Also, the VG1 position 157, which may have the mutation E157Q, does not appear to influence the INSTI therapy but is selected in treated patients (Charpentier et al., 2018). In VG1 two resistance-related positions (114 and 121) had zero entropy in both datasets, probably because the resistance mutation H114Y is rare (Goethals et al., 2008), and F121Y is rarely selected *in vivo* by RAL (Cavalcanti et al., 2012). Moreover, the VG1 resistance-related positions 51, 66, 95, 114, 121, 128, 142 and 149 showed entropies between zero and 0.1 in both datasets, showing a minimal degree of variability in the datasets of treated and non-treated patients. Interestingly, in Figure 1, it is possible to see five resistance-related positions grouped with H_RAL_ greater than 0.5 in VG1, these positions 74, 151, 163, 230, and 232.

The analysis of the Shannon entropies shows that VG3 bears the most important resistance hotspots. Also, all the positions involved in the three main resistance pathways are in VG3, showing that clustering the sequence positions by their entropies can be used to infer the selective pressure. Nevertheless, the use of entropy correspondence between both datasets as indicative for selective pressure does not seems to be sensitive to positions that have mutations with minor effects in INSTI therapy.

### HIV-1 Integrase Structure

**FIGURE 2:**
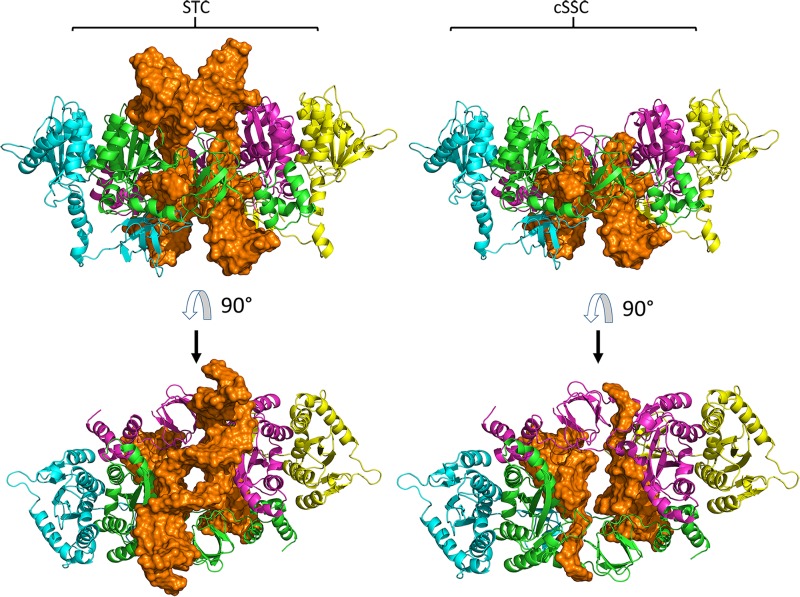
Models of the STC and cSSC. Chains A and C are close to the vDNA and shown respectively in green and magenta, while chains B and D are respectively represented in blue and yellow, DNA molecule is shown in orange. Both models are shown in front **(first row)** and top view **(second row)**.

**FIGURE 3:**
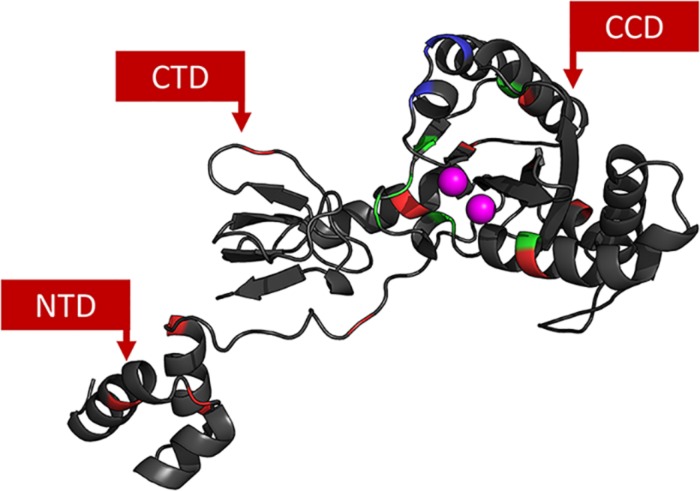
IN chain A. Each residue is colored according to its VG: VG1 in black, VG2 in red, VG3 in green and V4 in blue. Active site Mg^2+^ ions are colored in magenta. It is possible to see that the closer to the CCD, the more variable the residues are.

The 3D models constructed for cSSC and STC showed ≈98% of the residues in the allowed and favored regions of the Ramachandran plot, and all other stereochemical properties measured by the PROCHECK server (Laskowski et al., 1993) for both models are compatible with the stereochemical properties of deposited structures with ≈1.5 Å resolution. The Root-mean-square deviation (RMSD) values of the STC and the cSSC when compared with the STC structure used as a template, were 1.6 and 1.5 Å, respectively, highlighting that there was no significant alteration of the structures when the tDNA was removed. Moreover, the comparison of the cSSC structure of the PFV IN (3OYA) to STC structure shows an RMSD of 4.2 Å, suggesting that the experimentally solved cSSC structure and STC have very similar spatial arrangements. The whole structures of the cSSC and the STC can be seen in Figure 2. Figure 3 shows the distributions of the variability groups along the chain A of the IN. VG1 and VG2 residues are distributed across the whole IN structure, while VG3 and VG4 can only be found in the CCD.

**FIGURE 4:**
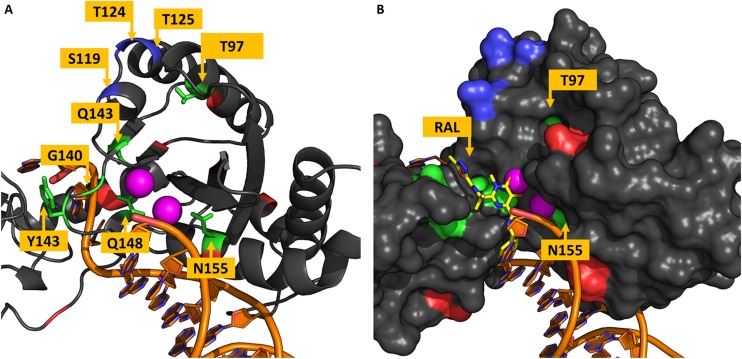
Residues in the vicinity of the active site. The chain A of the IN tetramer is shown in panels **(A,B)**. Residues are colored based on their variability groups; DNA molecule is shown in orange and Mg^2+^ ions in magenta. **(A)** Shows the distribution of residues around the active site, all residues from VG3 are located in this region, as well as all residues in VG4. **(B)** RAL molecule from the PFV intasome structure is shown in yellow sticks along with the IN Van der Waals surface; it is visible that N155 has no direct contact with RAL and is behind the Mg^2+^ ions. While T97 is ≈15 Å far from the closest RAL atom.

Residues in the CTD are shown in Figure 4A. All VG3 residues are located in the surroundings of the active site, as already known. To understand how RAL supposedly interacts with the IN, the PFV IN intasome bound to RAL was superimposed to the cSSC model. Figure 4B shows a superposition of the PFV IN intasome bound to RAL with the cSSC model. The superposition of the inhibitor molecule to the vDNA suggests that the RAL binding requires a conformational change in the terminal nucleotide of the vDNA strand – which is in an open state in the PFV IN. Thus, RAL probably binds by an induced-fit mechanism. The position T97 – that may bear the accessory mutation T97A – is ≈15 Å away from the closest RAL atom. This observation highlights the fact that T97A does not play a role impairing RAL binding but somehow compensates for the impacts of mutations Y143RC (Reigadas et al., 2011) and N155H (Malet et al., 2009; Canducci et al., 2010) and Q148H + G140S (Seki et al., 2015). Similar behavior is observed in residue N155, which has no direct contact with the RAL molecule. However, in this case, the mutation N155H is not an accessory mutation and the mechanism by which it causes resistance stays unclear. Grobler et al. (2008) speculated that the mutation N155H disrupts the coordinates of the Mg^2+^ ions in the active site. Crystallization of an equivalent mutant of the PFV IN with RAL molecules showed that only one Mn^2+^ ion could be found in the active site (Hare et al., 2010), the authors discussed the possibility that a stronger interaction of the terminal adenine of the vDNA with the histidine in position 155 could impair the induced-fit mechanism.

**FIGURE 5:**
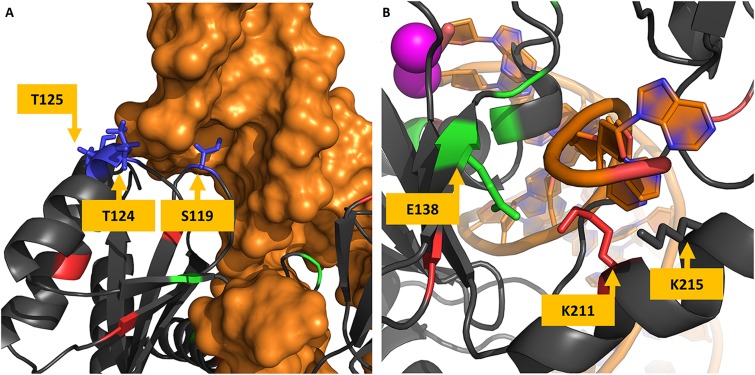
Protein-DNA interfaces in cSSC and STC. Panel **(A)** shows the structure of the STC chain A and the tDNA. The hypervariable residues S119, T124, and T125 are shown to interact with the tDNA (in orange), S119 side-chain is shown inside the minor groove of the DNA molecule. Panel **(B)** shows residues of the chain A of the IN cSSC colored by the VG they belong, E138, K211, and K215 are shown to interact with the terminal of the DNA strand that does not goes through the strand transfer reaction.

All the VG4 residues are located in the same region. Initially, the concentration of highly variable positions in a single region could be thought as a coincidence; however, as Figure 5A shows, the residue S119 has its side chain located inside the tDNA minor groove, while residues T124 and T125 also seem to have some minor interactions with the tDNA molecule. S119R is known as a resistance-related mutation and enhances primary resistance mechanisms (Hachiya et al., 2015). Moreover, the neighbor residue G118 can also be mutated to arginine in treated patients, as mentioned before. This observation raises the possibility that this region is involved in protein-DNA interaction, and maybe its high variability can be explained by some adaptation mechanism for DNA recognition. It is known that arginine residues are commonly found in interfaces between proteins and DNA minor grooves (Rohs et al., 2009), it could be possible that mutation S119R, as well as G118R, enhances the DNA binding, and therefore acts as a compensatory mechanism for primary resistance mutations.

A previous study that investigated DNA recognition did not test variations in positions S119, T124, and T125 (Chen et al., 2006); however, this study was carried out before the STC structure was determined, and the exact orientation of the tDNA was not known yet. Another study acknowledged the variations in this region and its structural localization (Ceccherini-Silberstein et al., 2009); but there was no conclusion about its role, possibly also because the STC structure was only determined 8 years later. A more recent analysis acknowledges the protein-DNA interaction role of T124 and states that the interaction is lost with mutation T124A (Rogers et al., 2018).

### Co-occurrence Network

The co-occurrence network was built with 68 mutations, considering only the mutation pairs with ξ indexes greater than 0.1. The frequencies of the mutations present in the network are shown by range in Table 1 (the frequency of each individual mutation within the network is shown in Supplementary Table S2). The resulting network had 68 nodes, 439 edges, and a density of 0.2. The resistance-related mutations found in the network were L74M, L74I, T97A, E138K, G140S, Y143R, Q148H, Q148R, N155H, E157Q, G163R, M50I, and S119R. Mutations E92Q and T66K – which are also resistance-related mutations – were not present in frequencies high enough to be considered in the network. The mutation pairs with ξ greater than 0.25 and their respective ξ values are depicted in Table 2.

**TABLE 1:** The frequency of IN mutations present in the co-occurrence network.

**Mutation**	**Frequency (% of isolates)**
V113I	90–99.9
I151V, V234L, V72I	80–89.9
L101I	50–59.9
V201I	40–49.9
V31I	30–39.9
D256E, **G140S**, **N155H**, **Q148H**, S17N, T124A, T125A	20–29.9
E10D, E11D, I208L, K14R, K156N, **M50I**, **Q148R**, S119P, S39C, T122I, T124N, T206S, T218S, **T97A**	10–19.9
A23V, A265V, D232E, D232N, D253E, D25E, D41N, D6E, **E138K**, **E157Q**, F181L, G163E, **G163R**, I135V, I203M, K160Q, K211R, K215N, K7R, L28I, L45V, **L74I**, **L74M**, M154L, N222K, Q216H, R20K, R284G, S119G, **S119R**, S230N, S283G, T112I, T125V, V165I, V234I, V32I, V79I, **Y143R**, Y227F	0–9.9

*All the mutations are shown by ranges of 10% frequency. It is possible to see that the all the resistance-related mutations (in bold) have frequencies lower than 30%. After filtering out the low frequency mutations, E92Q and T66K could not be found in the co-occurrence network.*

**TABLE 2:** Mutation pairs and their respective ξ values.

**Mutation pair**		**ξ**
G140S	Q148H	0.47
E157Q	K160Q	0.47
V113I	V234L	0.45
I151V	V113I	0.45
I151V	V234L	0.44
V113I	V72I	0.43
I151V	V72I	0.42
V234L	V72I	0.41
L101I	V72I	0.38
L101I	V113I	0.36
L101I	V234L	0.35
I151V	L101I	0.35
S119R	T97A	0.34
V113I	V201I	0.32
S119G	T122I	0.31
V201I	V72I	0.30
V201I	V234L	0.30
S119P	T122I	0.29
I151V	V201I	0.29
I203M	S119G	0.27
G163R	V79I	0.27
M154L	V165I	0.26
I203M	V234I	0.26
D253E	I203M	0.26
K215N	N222K	0.25
E138K	Q148R	0.25
D6E	E10D	0.25

*The table shows all the mutation pairs with ξ values greater than 0.25. The pair with the highest ξ value is G140S-Q148H, a known RAL-resistant combination.*

**FIGURE 6:**
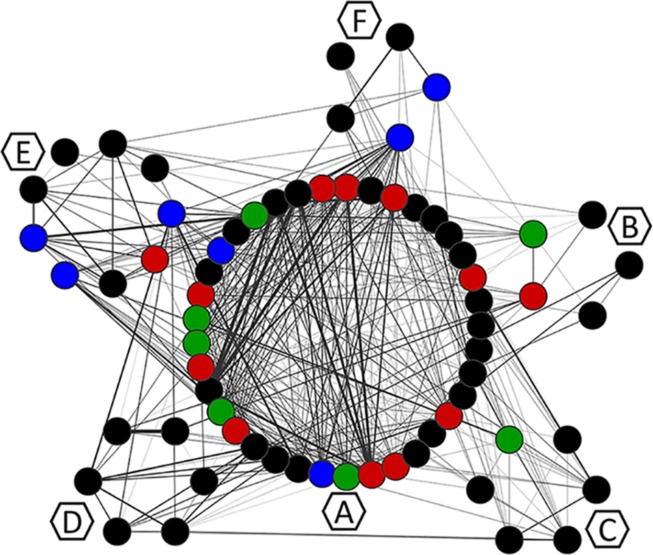
Mutation co-occurrence network. Each node corresponds to a mutation and is colored according to the VG of the position in which it occurs. Edges represent ξ indexes, and the higher the ξ value, the thicker the edge. Hexagons identify the MCL clusters to which residues were assigned with MCL. Residues are visually grouped according to their MCL cluster.

**FIGURE 7:**
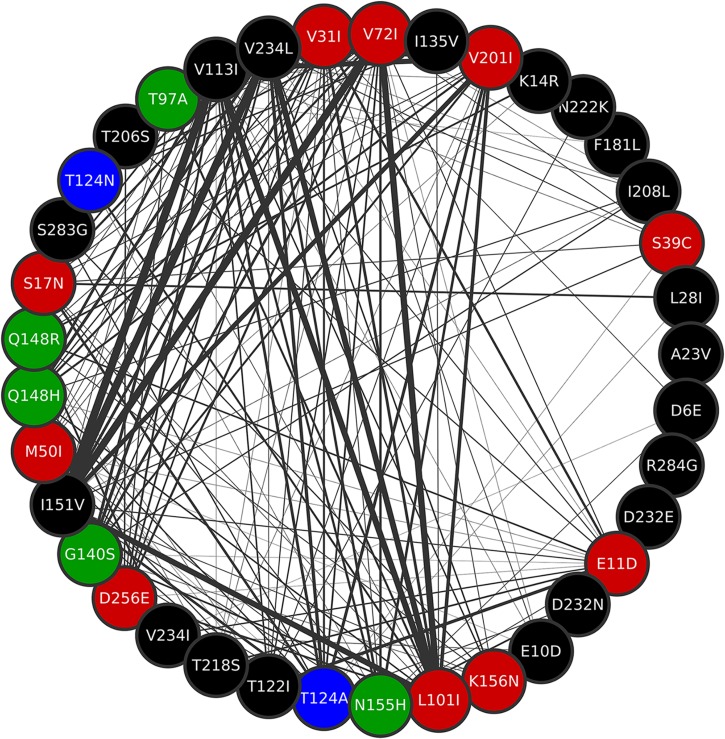
Cluster A of the co-occurrence network. Mutations Q148R, Q148H, G140S, N155H, and T97A are all contained in this cluster. All mutations in position T124 are also in cluster A. Thicker edges depict higher ξ values.

**FIGURE 8:**
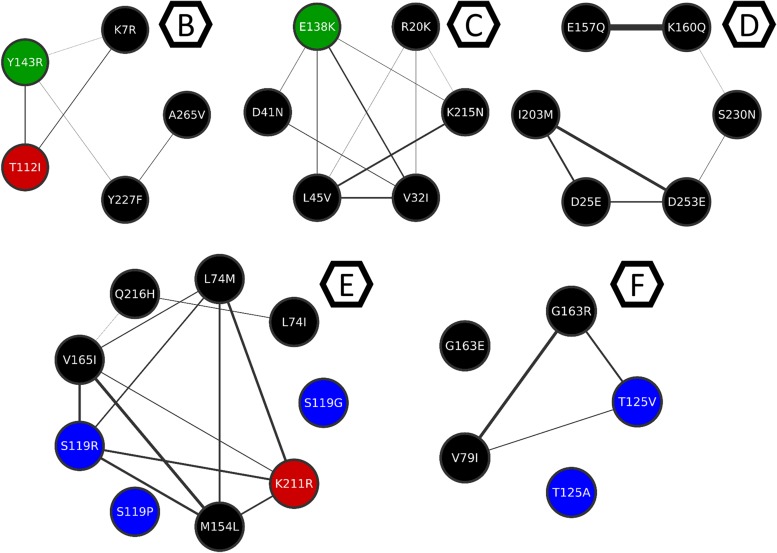
Clusters B to F from the mutation co-occurrence network. Cluster B, C, D, E, and F are depicted here for better understanding of the intra-cluster co-occurrences, and only the intra-cluster connections are shown. Thicker edges depict higher ξ values.

The MCL clustering divided the network into six clusters, namely, co-occurrence clusters A, B, C, D, E, and F (Figure 6). The clusters show different sizes. The major cluster (A), contains two mutations in the main resistance-related positions (Q148H and N155H), as well as the major resistance mutation G140S. While Y143R –a mutation in another of the three main resistance-related positions – is in cluster B. This is in agreement with the fact that resistance pathways are mutually exclusive, and the N155H pathway is more likely to be further converted into the Q148HKR variants than into Y143CRH (Fransen et al., 2009). It also shows that Y143R has its own set of co-occurring mutations. The mutations that co-occur with Q148R, Q148H, N155H, Y143R, E138K, and T97A are shown in Table 3. The detailed information about all the clusters can be seen in Figures 7, 8.

**TABLE 3:** Adjacent nodes of VG3 mutations.

**Mutation**	**Adjacent nodes in the network**
Q148R	D256E, D25E, D41N, E11D, E138K, I151V, I203M, K14R, K156N, K215N, S119G, T122I, T125A, T206S, T218S, V113I, V201I, V234I, V32I, V72I, V79I
Q148H	D256E, E11D, G140S, I151V, I208L, L101I, S283G, T122I, T124A, T125A, V113I, V201I, V234L, V31I, V72I
N155H	D232N, D6E, E10D, E11D, I151V, K156N, L101I, M50I, S119P, S17N, T112I, T122I, T124A, T124N, V113I, V201I, V234L, V31I, V72I
Y143R	E157Q, K7R, L74M, Q216H, S17N, T112I, T124N, T125A, T125V, T206S, T218S, T97A, V31I, Y227F
E138K	D41N, G163E, K14R, K156N, K215N, L45V, N222K, Q148R, R284G, V32I, V79I
T97A	D256E, F181L, I135V, I151V, K211R, L101I, L74M, M154L, S119P, S119R, S17N, T124N, T125A, T206S, V113I, V165I, V234L, V31I, V72I, Y143R

*The table depicts the VG3 mutations and their adjacent nodes in the network. The differences between the major pathways (Y143R, Q148R/H, and N155H) are clear when their adjacent nodes in the network are compared, highlighting the proximity between the N155H and the Q148HR pathways.*

It is also possible to see that except for Y143R and E138K, all the mutations on VG3 positions are in cluster A (Figure 6), which means they either co-occur or share co-occurring partners. Other resistance-related mutations found in cluster A, are D232N and M50I – both involved in increased resistance to DTG (Quashie et al., 2012; Tsiang et al., 2016). Cluster A also displays the mutations T124N and T124A, found VG4 positions.

The resistance-related mutations Y143R and E138K are populated in their own clusters, respectively B and C, which are populated mostly by mutations in low-entropy positions, and no other resistance-related mutation. Cluster D, on the other hand, has E157Q, which is a resistance-related mutation with minimal effects on RAL, and occurs in a VG1 position. Cluster D also has S230N, which is not associated with resistance to RAL; however, another mutation in this position (S230R) is associated with resistance to DTG (Pham et al., 2018). Moreover, within this cluster, mutation S230N co-occurs with K160Q, which takes place in a DNA-anchoring lysine (Chen et al., 2006).

Cluster E is a diverse cluster, with mutations from different variability groups. Position L74 has two mutations within this cluster: L74M and L74I, both are described as resistance-related, also described as having minimal effects in INSTI therapy, as previously said. Mutations S119RPG are also present in this cluster. S119R seems to be selected in RAL treated patients, and alone seems to have only minor effects in INSTIs (Hachiya et al., 2015), and within cluster E, S119R co-occurs with L74M. As mentioned above, S119R may have implications in DNA binding.

Cluster F also has mutations T125V and T125A, which are VG4 positions. The latter co-occurs with G163R, which is an accessory mutation to the N155H pathway selected in patients receiving RAL (Charpentier et al., 2008; Cooper et al., 2008).

E138K co-occurs with three mutations of lysine residues, one of them in K156 (K156N). Along with K159 and K160, K156 is known for having important roles in DNA binding and may affect enzyme activity (Jenkins et al., 1997; Chen et al., 2006; Krishnan et al., 2010). Other mutations that co-occur with E138K are K215N and K14R. K215 is close to the DNA strand that does not participate in the reaction, as well as K211. Interestingly, on the other side of the strand is E138 (Figure 5B). It is known that lysine residues are usually found in these regions and participate in the protein-DNA recognition process (Luscombe et al., 2001). Therefore, mutation E138K could be compensating for K215N, given their co-occurrence and structural proximity. By the observed co-occurrence, E138K could also be compensating for K156N. Another example of this possible compensatory mechanism is the co-occurrence of K215N and N222K. We believe that these can be examples of adaptation mechanisms that maintain the DNA binding activity of the enzyme.

## Conclusion

In this work, we were able to show how variable are the IN positions both in drug-naïve and RAL-treated patients. It is clear that the entropies of positions in both datasets are highly correlated, and that residues that have low entropies in drug-naïve patients while having high entropies in RAL-treated ones are mainly major resistance-related positions.

Analysis of the structure of cSSC revealed that the coordinates of residues T97 and N155 do not directly explain their implications in resistance. The position of the RAL molecule when superimposed in the IN structure shows that it possibly binds through an induced fit mechanism. It is reinforced when looking at the different conformation that the RAL-bonded PFV IN terminal nucleotide displays. Both T97 and N155 are far from the RAL molecule when the RAL-bond PFV integrase is superimposed with the cSSC structure. These data indicate a non-obvious resistance mechanism, possibly by a rearrangement of the Mg^2+^ ions or by impairment of the induced-fit mechanism. More studies on the structure and dynamics of the complex are needed to uncover how these mutations contribute to resistance.

The STC structure showed that residues S119, T124, and T125 are in the vicinity of the tDNA and possibly play a role in its recognition. S119R could have a substantial impact on how the IN binds to the tDNA, as well as G118R.

The co-occurrence network showed that N155H and Q148HR pathways share more co-occurring mutations with each other than with Y143R, which is consistent with the frequency by which N155H is further converted into Q148HR or Y143CRH (Fransen et al., 2009). The clustering of the co-occurrence network showed that mutation E138K is possibly involved in maintaining DNA-recognition function when other DNA-anchoring lysine residues are mutated. The same pattern was seen in N222 mutating to lysine when K215 is mutated to asparagine.

The exploration of mutational patterns can help us understand how the IN adapts during treatment; how mutations compensate for the absence of other important residues; and which regions are allowed to be variable. This kind of knowledge is fundamental to search for new therapeutic options and rethink the currently used drugs. The present work corroborates observations of previous studies that correlated analysis of structures and mutations, mainly in what concerns the degree of variation (Ceccherini-Silberstein et al., 2009) and roles of certain residues (Rogers et al., 2018). Also, here we contributed with new information regarding the relationship between different mutations, showing new interpretations for their possible roles.

## Data Availability

The raw data supporting the conclusions of this manuscript will be made available by the authors, without undue reservation, to any qualified researcher.

## Author Contributions

LM and AG designed the research and wrote the manuscript. MG conceptualized the co-occurrence network. LM carried out the calculations and analysis.

## Conflict of Interest Statement

The authors declare that the research was conducted in the absence of any commercial or financial relationships that could be construed as a potential conflict of interest.
