# Supplementary material for: Raltegravir-Induced Adaptations of the HIV-1 Integrase: Analysis of Structure, Variability, and Mutation Co-occurrence
Source: Front Microbiol. 2019 Sep 3;10:1981. doi: 10.3389/fmicb.2019.01981 (PMC6733956; doi:10.3389/fmicb.2019.01981)
Supplement: Supplementary file 2 [file Table_1.docx]

**SUPPLEMENTARY TABLE 1.** Frequency of the mutations in the RAL-treated patients’ dataset

| Mutation | Frequency (%) |
| --- | --- |
| V113I | 91.77 |
| V234L | 89.24 |
| I151V | 86.71 |
| V72I | 80.38 |
| L101I | 56.33 |
| V201I | 47.47 |
| V31I | 30.38 |
| T124A | 29.75 |
| T125A | 29.75 |
| G140S | 28.48 |
| Q148H | 26.58 |
| N155H | 25.32 |
| D256E | 21.52 |
| S17N | 21.52 |
| K156N | 17.09 |
| T122I | 17.09 |
| E11D | 15.82 |
| T124N | 15.82 |
| T206S | 15.82 |
| S119P | 15.19 |
| M50I | 14.56 |
| T97A | 14.56 |
| S39C | 13.92 |
| Q148R | 12.03 |
| I208L | 11.39 |
| T218S | 11.39 |
| E10D | 10.76 |
| K14R | 10.76 |
| D232N | 9.49 |
| S119R | 9.49 |
| S283G | 9.49 |
| I135V | 8.86 |
| T112I | 8.86 |
| V32I | 8.86 |
| D253E | 8.23 |
| S230N | 8.23 |
| L28I | 7.59 |
| N222K | 7.59 |
| R20K | 7.59 |
| S119G | 7.59 |
| V165I | 7.59 |
| Y143R | 7.59 |
| D6E | 6.96 |
| D25E | 6.33 |
| I203M | 6.33 |
| A23V | 5.70 |
| A265V | 5.70 |
| E138K | 5.70 |
| K211R | 5.70 |
| V234I | 5.70 |
| D41N | 5.06 |
| E157Q | 5.06 |
| G163E | 5.06 |
| G163R | 5.06 |
| K215N | 5.06 |
| K7R | 5.06 |
| L74M | 5.06 |
| Q216H | 5.06 |
| D232E | 4.43 |
| F181L | 4.43 |
| K160Q | 4.43 |
| L45V | 4.43 |
| L74I | 4.43 |
| M154L | 4.43 |
| R284G | 4.43 |
| T125V | 4.43 |
| V79I | 4.43 |
| Y227F | 4.43 |
| A21S | 3.80 |
| A21T | 3.80 |
| E138A | 3.80 |
| I220L | 3.80 |
| Q221S | 3.80 |
| S24N | 3.80 |
| T112A | 3.80 |
| A23S | 3.16 |
| G140A | 3.16 |
| G193E | 3.16 |
| L45I | 3.16 |
| M154I | 3.16 |
| M275V | 3.16 |
| R269K | 3.16 |
| S24G | 3.16 |
| T112V | 3.16 |
| V37I | 3.16 |
| Y143C | 3.16 |
| D167E | 2.53 |
| D278E | 2.53 |
| E138D | 2.53 |
| E13D | 2.53 |
| E92Q | 2.53 |
| G277C | 2.53 |
| G277R | 2.53 |
| I208M | 2.53 |
| K111T | 2.53 |
| K219N | 2.53 |
| K7Q | 2.53 |
| S119T | 2.53 |
| S230R | 2.53 |
| A205S | 1.90 |
| D270H | 1.90 |
| D286N | 1.90 |
| E212A | 1.90 |
| I200L | 1.90 |
| I268L | 1.90 |
| K111R | 1.90 |
| K136N | 1.90 |
| K136Q | 1.90 |
| K188R | 1.90 |
| N254Q | 1.90 |
| S147G | 1.90 |
| S195C | 1.90 |
| S255N | 1.90 |
| S255R | 1.90 |
| S39N | 1.90 |
| S57G | 1.90 |
| V126M | 1.90 |
| V54I | 1.90 |
| V77A | 1.90 |
| Y143S | 1.90 |
| A196P | 1.27 |
| A23G | 1.27 |
| A91T | 1.27 |
| D270E | 1.27 |
| D270N | 1.27 |
| D278G | 1.27 |
| D279G | 1.27 |
| D6N | 1.27 |
| E35Q | 1.27 |
| G106A | 1.27 |
| G140C | 1.27 |
| G163Q | 1.27 |
| G59E | 1.27 |
| I84M | 1.27 |
| K103R | 1.27 |
| K173R | 1.27 |
| L45Q | 1.27 |
| M50L | 1.27 |
| M50T | 1.27 |
| P142T | 1.27 |
| Q221N | 1.27 |
| Q95K | 1.27 |
| Q9H | 1.27 |
| V234H | 1.27 |
| V281M | 1.27 |
| Y143G | 1.27 |
| Y143K | 1.27 |
| A128T | 0.63 |
| A169S | 0.63 |
| A169T | 0.63 |
| A169V | 0.63 |
| A205T | 0.63 |
| A49P | 0.63 |
| A49S | 0.63 |
| D229G | 0.63 |
| D232S | 0.63 |
| D253V | 0.63 |
| D279H | 0.63 |
| D3E | 0.63 |
| D6T | 0.63 |
| E10A | 0.63 |
| E10G | 0.63 |
| E170A | 0.63 |
| E212K | 0.63 |
| E212L | 0.63 |
| G163T | 0.63 |
| G272E | 0.63 |
| G70N | 0.63 |
| G70R | 0.63 |
| G82E | 0.63 |
| H16P | 0.63 |
| H171L | 0.63 |
| H171Q | 0.63 |
| I161F | 0.63 |
| I161T | 0.63 |
| I162V | 0.63 |
| I182V | 0.63 |
| I204V | 0.63 |
| I217V | 0.63 |
| I220M | 0.63 |
| I220V | 0.63 |
| I36V | 0.63 |
| I60M | 0.63 |
| I84V | 0.63 |
| I89V | 0.63 |
| K111Q | 0.63 |
| K127R | 0.63 |
| K136T | 0.63 |
| K156R | 0.63 |
| K211N | 0.63 |
| K211Q | 0.63 |
| K215S | 0.63 |
| K219Q | 0.63 |
| K264R | 0.63 |
| K34R | 0.63 |
| K7D | 0.63 |
| K7E | 0.63 |
| L158I | 0.63 |
| L28M | 0.63 |
| L2W | 0.63 |
| L63I | 0.63 |
| L74V | 0.63 |
| M22I | 0.63 |
| M22L | 0.63 |
| M22R | 0.63 |
| N117T | 0.63 |
| N254G | 0.63 |
| N254S | 0.63 |
| N27H | 0.63 |
| N27S | 0.63 |
| P30A | 0.63 |
| P30S | 0.63 |
| Q148K | 0.63 |
| Q148N | 0.63 |
| Q168R | 0.63 |
| R187K | 0.63 |
| R224Q | 0.63 |
| R231K | 0.63 |
| R263K | 0.63 |
| S123G | 0.63 |
| S17T | 0.63 |
| S195T | 0.63 |
| S24A | 0.63 |
| S24D | 0.63 |
| S255G | 0.63 |
| S255K | 0.63 |
| S283N | 0.63 |
| S39R | 0.63 |
| T112K | 0.63 |
| T112M | 0.63 |
| T125P | 0.63 |
| T210S | 0.63 |
| T218I | 0.63 |
| T66K | 0.63 |
| T93N | 0.63 |
| V113M | 0.63 |
| V126L | 0.63 |
| V176L | 0.63 |
| V259I | 0.63 |
| V31M | 0.63 |
| V75M | 0.63 |
| Y143H | 0.63 |
| Y194C | 0.63 |

The table depicts the frequencies of each individual mutation present in the RAL-treated patients’ dataset.

**SUPPLEMENTARY TABLE 2.** Frequency of the mutations in the co-occurrence network

| Mutation | Frequency (% of isolates) |
| --- | --- |
| V113I | 91.8 |
| V234L | 89.2 |
| I151V | 86.7 |
| V72I | 80.4 |
| L101I | 56.3 |
| V201I | 47.5 |
| V31I | 30.4 |
| T124A | 29.7 |
| T125A | 29.7 |
| G140S | 28.5 |
| Q148H | 26.6 |
| N155H | 25.3 |
| D256E | 21.5 |
| S17N | 21.5 |
| K156N | 17.1 |
| T122I | 17.1 |
| E11D | 15.8 |
| T124N | 15.8 |
| T206S | 15.8 |
| S119P | 15.2 |
| M50I | 14.6 |
| T97A | 14.6 |
| S39C | 13.9 |
| Q148R | 12.0 |
| I208L | 11.4 |
| T218S | 11.4 |
| E10D | 10.8 |
| K14R | 10.8 |
| D232N | 9.5 |
| S119R | 9.5 |
| S283G | 9.5 |
| I135V | 8.9 |
| T112I | 8.9 |
| V32I | 8.9 |
| D253E | 8.2 |
| S230N | 8.2 |
| L28I | 7.6 |
| N222K | 7.6 |
| R20K | 7.6 |
| S119G | 7.6 |
| V165I | 7.6 |
| Y143R | 7.6 |
| D6E | 7.0 |
| D25E | 6.3 |
| I203M | 6.3 |
| A23V | 5.7 |
| A265V | 5.7 |
| E138K | 5.7 |
| K211R | 5.7 |
| V234I | 5.7 |
| D41N | 5.1 |
| E157Q | 5.1 |
| G163E | 5.1 |
| G163R | 5.1 |
| K215N | 5.1 |
| K7R | 5.1 |
| L74M | 5.1 |
| Q216H | 5.1 |
| D232E | 4.4 |
| F181L | 4.4 |
| K160Q | 4.4 |
| L45V | 4.4 |
| L74I | 4.4 |
| M154L | 4.4 |
| R284G | 4.4 |
| T125V | 4.4 |
| V79I | 4.4 |
| Y227F | 4.4 |

The table shows the frequencies of each individual mutation found within the co-occurrence network.

**Dataset.csv documentation:**

The dataset.csv file contains all the isolates used in the study, as well as their information and sequence of the integrase region, it is a reduced version of the dataset found in the HIV drug resistance database. The file has 9 columns:

RefID: Reference identifier of the study. The reference list can be found at <https://hivdb.stanford.edu/pages/geno-rx-datasets.html>

PtID: Patient ID
Isolatename: Isolate identifier
Region: Region of sampling

Year:Year of sampling
INIlist: List of integrase inhibitors used in the treatment regimen

AssecionID: Genbank accecssion number
